# Data on retail price differential between organic and conventional foods

**DOI:** 10.1016/j.dib.2019.104641

**Published:** 2019-10-11

**Authors:** Shahidul Islam, Constantin Colonescu

**Affiliations:** MacEwan University, 10700 – 104 Avenue, Edmonton, Alberta T5J 4S2, Canada

**Keywords:** Organic foods, Comparative prices, Conventional consumers, Price differential

## Abstract

The objective of this dataset is to find out retail price differences between organic and conventional food items. Organic foods are often considered healthier and better quality than conventional foods and are sold at premium prices. However, first-hand data on retail price levels to substantiate that argument is meager. With a view to filling up that gap, we collected retail prices for pairs of conventional and organic food items in three supermarket chains (Save On Foods, Superstore, and Sobeys) in Edmonton, Alberta, for seven consecutive weeks in spring 2011. We find that the average prices significantly vary among supermarkets and among different food groups. Organic food prices show a different pattern than conventional food prices.

**Table:** Specifications Table

**Subject**	Economics and Econometrics, Marketing
**Specific subject area**	Retail price premium for organic foods
**Type of data**	Table Figures
**How data were acquired**	Periodic visits to retail supermarkets to record price data
**Data format**	Raw Analyzed
**Parameters for data collection**	Price per selling unit (package, container, pound, liter, etc.) were recorded for each item and then converted to standard units for comparison. Over 150 individual food items were grouped into 17 categories.
**Description of data collection**	Price data were recorded from three different supermarkets for a period of seven consecutive weeks. Each store was visited at the same time of the day and of the same day of the week.
**Data source location**	Data are provided as a supplementary file with this article.
**Data accessibility**	With the article as a supplementary file. (Islam, 2011)

**Table:** 

**Value of the Data** • This is a primary dataset on retail prices of organic and conventional food items from supermarkets. This dataset not only gives information about the differences in prices charged for organic foods versus conventional foods, but also offers a micro-level information on such differences for each food item. • Any researcher who wants to conduct a comparative retail price analysis between organic and conventional foods will find this dataset useful. Most such data are available on whole-sale prices only. • This dataset offers the opportunity to explore more insights in the price premiums for organic foods. • Researchers in organic food marketing may further analyse this data and develop further experiments on price differences in specific food categories. For example, further experimentation can be developed on why certain price differences exist on certain food items. • To our knowledge, this retail-level price data is the first of its kind, at least in Canada.

## 1 Data

The raw data is in the supplementary file [2]. Organic food prices often differ from conventional food prices [[3], [4]]. Prices we report here are all per unit. While a more informative measure of price levels would be to calculate a quantity-weighted price index, we report the per-unit nominal prices and calculate the mean prices using simple arithmetic average since we do not have quantity data for each product. Our method, however, is still valid for comparing prices between organic and conventional across stores and for different food groups as prices are in common units.

Table 1 shows the variables in the dataset; *Week* is one of the seven weeks, *Category* is one of the 17 food categories defined later, *Store* is one of the three stores, *Type* is either organic or conventional, *Price* is recorded in nominal Canadian dollar as labelled on the shelf, and *Item* is the description of the actual food item for which price is recorded.

**Table 1:** A sample of the price dataset.

Week	Category	Store	Type	Price	Item
7	1	1	conventional	$0.19	apples red delicious
7	1	1	conventional	$0.24	apples granny smith
7	1	1	conventional	$0.24	apples gala
7	1	1	conventional	$0.17	bananas
7	1	1	conventional	$0.18	oranges
7	1	1	conventional	$0.21	grapes
6	1	1	conventional	$0.19	apples red delicious
6	1	1	conventional	$0.24	apples granny smith
6	1	1	conventional	$0.24	apples gala
6	1	1	conventional	$0.17	bananas
6	1	1	conventional	$0.18	oranges
6	1	1	conventional	$0.21	grapes
5	1	1	conventional	$0.26	apples red delicious
5	1	1	conventional	$0.24	apples granny smith
5	1	1	conventional	$0.24	apples gala

There are 2814 observations (1407 price pairs, organic-conventional foods). Table 2 summarizes average nominal price differentials between organic and conventional food items in three supermarkets.

**Table 2:** Sample sizes and price means by store and type.

Store	Obs	Mean price conventional	Mean price organic
1	427	0.77	1.44
2	315	0.84	1.25
3	665	1.00	1.53

**Fig. 1:**
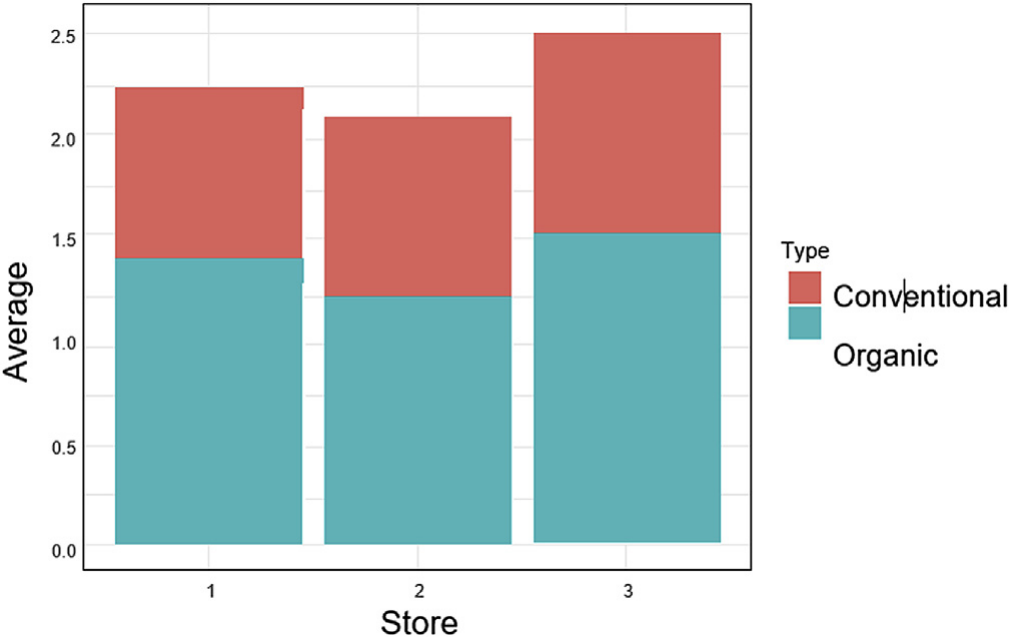
Comparative means by food type and store.

Fig. 1 depicts the average prices by store and type; it suggests that average price levels across stores are different, which, if confirmed by equality of means tests, would question the competitiveness of food markets in Edmonton. If the test comes out significant, it would be interesting to explore the possible factors that contribute to this difference in average prices. Speculating about the causes of such differences is, however, beyond the scope of this report.

Fig. 1 also suggests that stores 1 and 3 seem to charge a higher markup on organic food than store 2. This conjecture, again, needs to be tested. Equality of means tests requires that the data be normally distributed, unless the samples are sufficiently large. Table 2 gives the sizes of the samples and the mean prices by store; the samples for conventional and organic food are equal at each store.

**Fig. 2:**
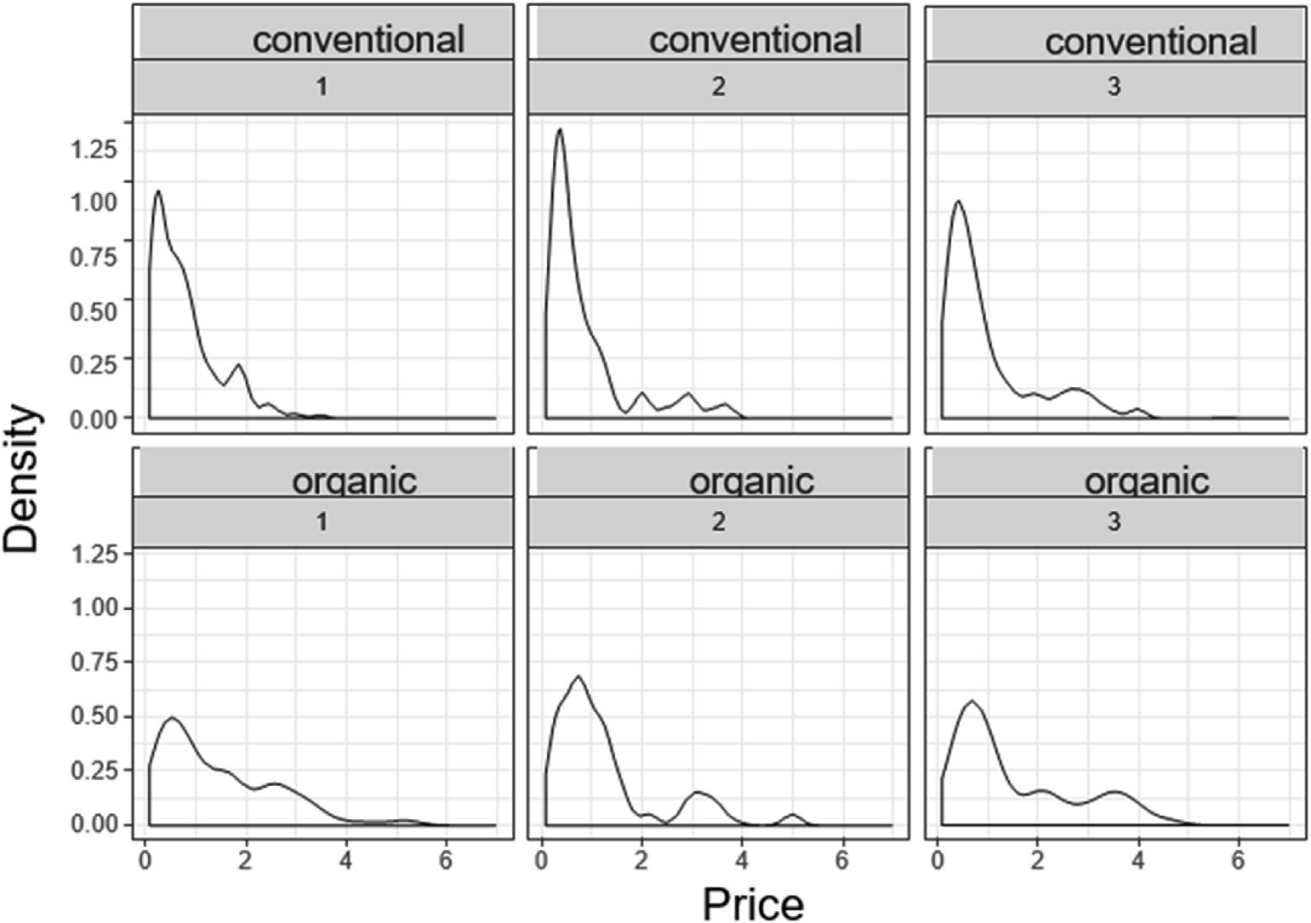
Density plots and prices by Type and Store.

Fig. 2 shows that the distributions of prices, both conventional and organic are strongly skewed to the right, which inspires us to use non-parametric equality-of-means tests. However, using parametric tests, such as the classical *t*-test, would probably still be acceptable, since our sample sizes are sufficiently large.

We use the Kruskal-Wallis test to check whether there are significant differences in organic food prices across the three stores. The test indicates a p-value of 0.013, which rejects the null hypothesis that the mean prices for organic food are equal in the three stores. Applying the same test to conventional food prices, we also see evidence that the prices are different, on average, in the three stores.

Inspecting the data and the relative averages of the prices of conventional vs. organic food show that these prices are different, with organic food showing the higher averages for each store. For accuracy, however, we test this supposition for each store. Testing this hypothesis requires some care, though, because the conventional vs. organic samples may not be independent: each observation in these samples is a pair of the prices of the same product in its conventional vs. organic version. The Wilcoxon Matched-Pairs Signed test is the appropriate tool for this purpose, which shows that organic food is more expensive than conventional food.

Table 3 presents the correlation coefficients (calculated by the Spearman method) between organic and conventional food prices in the three supermarkets. While there is some difference in prices among stores, more differences exist between the prices of the two categories of products.

**Table 3:** Correlation coefficients between organic and conventional food prices by stores.

Store 1	Store 2	Store 3
0.851	0.831	0.822

## 2 Experimental design, materials, and methods

Five conventional grocery stores, Sobeys, Save On Foods, Superstore, Safeway and Walmart in Edmonton, Alberta, Canada, were approached for allowing us to record the weekly prices of certain organic and their conventional counterpart food items. The stores were assured that the raw prices or comparative prices of individual food items among different stores would not be published or disclosed to anyone. Only the normalized and aggregate prices of different food groups would be reported. After repeated requests and with enough assurance that the findings would only be used for research purposes and would not be released to anyone, Safeway and Walmart refused to cooperate.

The retail price data for selected organic food items along with their conventional counterparts were recorded from three retail grocery stores for a period of seven consecutive weeks. Such price data were organized into 17 different food categories. The food categories and the items included in those categories are presented below:

1. Fresh Fruits: Apples, bananas, oranges, grapes, pears, grapefruit, kiwi, cantaloupe, honeydew melon, water melon, strawberries, blueberries, raspberry, mangoes, etc.
2. Fresh Vegetables: Carrot mini, onions, sweet potatoes, cauliflower, celery, romaine lettuce, avocado, white mushroom, tomatoes, grape tomatoes, acorn squash, garlic, yams, red potatoes, broccoli, beet bunch, peeled carrots, cilantro, head lettuce, green pepper, yellow pepper, zucchini, English cucumber, baby carrots, green onion, etc.
3. Dry Snacks and Crackers: Crackers, chocolate chips, walnut crumbs, cashews, pumpkin seeds, sunflower seeds, popcorn, crystalized ginger, sultan raisins, chocolate almonds, soy nuts, trail mix, fruit and nut mix, banana chips, Special K vanilla almond, Kellogg's rice bar, Planters peanut, granola bars, etc.
4. Rice, Wheat, and Pasta: Pasta and penne, all forms of rice and flour, etc.
5. Breakfast Cereals: Hot and cold cereals, bread, pancake mix, waffles, instant oatmeal, etc.
6. Sugar, Syrup, and Honey: Brown and white sugar, syrups, honey
7. Tea and Coffee: All brands of tea and coffee, bulk and packed, etc.
8. Canned Fruits and Vegetables: Canned tomatoes, beans, corn, peas, etc.
9. Ready-to-eat Canned Food: Soups, broths, burritos, etc.
10. Jam, Jelly, and Spread: Peanut butter, herb paste, jam, jelly, etc.
11. Salad Dressings, Ketchups and Sauces: Pasta sauce, ketchups, all forms and brands of salad dressings, pickles, etc.
12. Milk and Dairy Products: Milk, cheese, buttermilk, butter, sour cream, ice cream, etc.
13. Eggs and Egg Products: Egg, Eggo, etc.
14. Juice and Beverages: Different fruit juices and soy beverages.
15. Oil and Vinegar: Different oils and vinegar
16. Fresh Meat: All forms of beef, mutton and chicken cuts.
17. Ready-made Pizza: Ready to eat pizza of all brands.

## Funding

This data collection process received funding from MacEwan University, Canada (Grant No. 00324).
